# From Enhancement to Elimination of Auxeticity by Inserting Multilayers of Diatomic Molecules into a Hard-Sphere Crystal

**DOI:** 10.3390/ma19153354

**Published:** 2026-08-06

**Authors:** Jakub W. Narojczyk, Krzysztof W. Wojciechowski, Konstantin V. Tretiakov

**Affiliations:** Institute of Molecular Physics, Polish Academy of Sciences, M. Smoluchowskiego 17, 60-179 Poznan, Poland; narojczyk@ifmpan.poznan.pl (J.W.N.); kww@ifmpan.poznan.pl (K.W.W.)

**Keywords:** auxetics, negative Poisson’s ratio, hard spheres, hard dumbels, inclusions, Monte Carlo simulations

## Abstract

Predicting how atomic-scale structural changes influence a system’s elastic properties is challenging in general. In this study, we examined how the reduction in the atomic degrees of freedom within layer inclusions embedded in a hard-sphere crystal affects its auxetic behaviour. This reduction was achieved by randomly pairing the neighbouring hard spheres into diatomic molecules called dumbbells. Specifically, we explored the effects of periodically introducing one-, two-, and three-layer inclusions of dumbbells on the crystal’s elastic properties. Our results show that inclusions of adjacent layers filled with dumbbells notably alter the elastic behaviour compared to systems with similar inclusions composed of spheres. We observed that by rigidly connecting neighbouring spheres to form diatomic molecules within inclusion layers, two opposing effects can be achieved. First, at slightly smaller diameters of the spheres forming dumbbells, we found an enhancement of auxetic properties in comparison with the system containing only hard spheres. Second, for systems with large sphere diameters forming dumbbells, we observed that the crystal’s auxetic properties were almost eliminated.

## 1. Introduction

Materials with specific physical properties drive modern technological advancements, alongside other materials with required elastic features. Auxetics [1,2] exemplify such materials with unusual elastic behaviour, as they expand transversely when stretched. This is contrary to typical materials like rubber, which shrink under tension. Poisson’s ratio (PR) [3] is a relevant physical parameter that describes this behaviour, defined as the negative ratio of transverse strain to longitudinal strain when the longitudinal stress changes infinitesimally [3]. Most materials exhibit a positive Poisson’s ratio; for example, rubber has about +0.5. According to elasticity theory, Poisson’s ratio can range from 1/2 to −1 in 3D isotropic materials [3]. Although negative values are predicted by elasticity theory for Poisson’s ratio, such materials were only manufactured in the late 1980s [4]. During that time, several thermodynamic models demonstrated this phenomenon [5]. A few years later, materials with a negative Poisson’s ratio became known as auxetics [6]. Over the past forty years, auxetics have attracted increasing interest due to their potential uses in medicine [7,8], sports [9,10], and other fields [11,12]. Among these, auxetic composites [13,14] also garner attention. Layered structures, which are central to this study, are extensively utilized in composite materials, which demonstrate advantageous physical, mechanical, and other traits across various fields [15]. These include applications in electronics [16], aircraft brake construction [17], and even as catalysts and adsorbents [18]. Inspired by composite materials, recent pioneering methods aim to design materials at the atomic level by modifying their microcrystalline structure [19]. Building on this concept, altering the microstructure allows the creation of materials with specific elastic properties, recognizing that such structures often display anisotropy in their elastic behaviour. For instance, the Poisson’s ratio of anisotropic crystals depends on the orientations of both the longitudinal and transverse directions relative to the crystalline axes. If the Poisson’s ratio is negative in all crystallographic directions, the material is considered auxetic. Conversely, a non-auxetic material is one where the Poisson’s ratio is never negative in any direction. Partial auxetics are materials that display a negative Poisson’s ratio in some directions while having zero or positive PR in others [20]. Notably, about 69% of cubic metals [21] are partial auxetics. The hard-sphere model, a fundamental model in liquids and condensed matter physics, is also partially auxetic in its face-centered cubic (FCC) crystalline phase. Numerous studies have examined the elastic properties of hard-sphere crystals [22,23,24,25,26,27,28]. Specifically, the FCC hard-sphere crystal shows a negative Poisson’s ratio in the $\left[110\right]\left[1\bar{1}0\right]$ crystallographic direction, measuring −0.054(9) at melting density [23] and −0.072(1) in the close packing limit [29].

So far, various systems with nanoinclusions have been studied. Research on nanochannel systems shows that arrays of parallel nanochannels can significantly improve auxetic properties [30]. Conversely, inclusions arranged in three mutually orthogonal nanochannel arrays eliminate auxetic behaviour [31]. Carefully choosing the number and orientation of nanochannel inclusions can produce interesting effects, such as increased crystal stiffness [32]. Earlier research suggests that predicting how nanoinclusions affect elastic properties is complex. For instance, introducing interconnected nanochannels in the [001] direction and (001) nanolayers removes auxetic properties from hard sphere crystals [33], which is surprising since these inclusions independently enhance auxetics [30,34]. Additional studies on layered inclusions show that adjusting their spatial arrangement can substantially boost auxetic responses in specific crystallographic directions [35]. A recent model of a crystal with nanolayer inclusions and an orthogonal array of nanochannels filled with a degenerate crystalline phase [36] of hard dumbbells revealed a notable reduction in Poisson’s ratio in the [110] direction, along with an overall increase in auxetic behaviour [37]. Nonetheless, the impact of limiting atomic degrees of freedom on elastic properties, particularly auxetic ones, in the presence of other types of inclusions remains an open question.

This work examines how reducing the degrees of freedom of atoms within layer inclusions inserted into the FCC crystal as (001) planes affects the material’s elastic properties. The goal is to determine the elastic properties of these modified crystals, where particles interact via a hard potential. Specifically, we explore how reducing atomic degrees of freedom in the layer inclusions affects the crystal’s auxetic behaviour. Our findings demonstrate that the structural modification studied significantly affects the auxeticity of the model system, effectively eliminating it. We believe that in the near future, this knowledge will be valuable for the design of new materials.

## 2. Models and Methods

### 2.1. Models

In this study, we analyze a model based on an FCC crystal composed of *N* spheres of diameter σ, which we also use as the unit length. The studied models are created by replacing spheres in one, two, or three adjacent layers with particles of a different size, $\sigma^{\prime }\neq \sigma$. These layers are orthogonal to the [001]-direction and are called inclusion layers, while the remaining spheres form the so-called matrix. All spheres within each inclusion are identical, resulting in a binary system of two particle types that differ only in size. All spheres in the system interact *via* a purely geometric hard potential of the form:

(1) $$\beta u_{ij}=\left\{\begin{array}{cc} \infty , & r_{ij}<\sigma_{ij},\\ 0, & r_{ij}\geq \sigma_{ij}, \end{array}\right.$$

where $r_{ij}$ is the distance between the centres of spheres *i* and *j*; $\sigma_{ij}$ is the sum of their radii, $\beta =1/(k_{B}T)$; $k_{B}$ is the Boltzmann constant; *T* is the temperature. This simple athermal interaction potential is a fundamental model in the theory of liquids and condensed matter physics [38,39]. It is particularly useful for studying the elastic properties of systems, because models with this interaction can exhibit a negative Poisson’s ratio. The hard-body interaction potential is sensitive to changes in the size of the interacting particles. The inclusion spheres have diameters $\sigma^{\prime }\neq \sigma$. As a result, the impact of inclusions will be examined in terms of the ratio $\sigma^{\prime }/\sigma$, which indicates the relative change in the diameters of the inclusion and matrix spheres.

**Figure 1:**
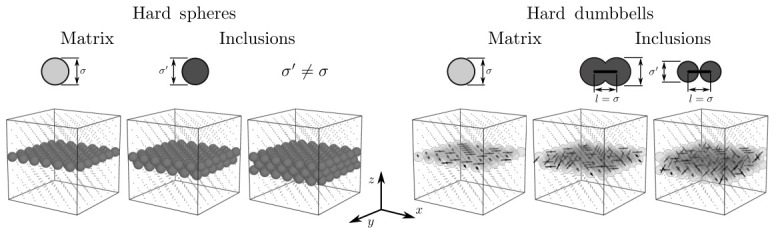
Models with one (L1), two (L2), and three (L3) inclusion layers. The top images display particle shapes—spheres and dumbbells. *l* is the distance between the centres of spheres that form the molecule and is further referred to as the length of the dumbbell. The light gray matrix spheres form the initial FCC crystal, and the dark grey inclusion spheres are randomly connected into dumbbell molecules. In the presented structures, the diameters of the matrix particles have been artificially reduced to highlight the structure of the inclusion and the specific (random) placement of dumbbells within the nanoinclusion layers.

Additionally, to examine how decreasing the number of atomic degrees of freedom impacts the system’s elastic properties, pairs of neighbouring spheres that form inclusion layers have been randomly connected by rigid bonds to form molecules (see Figure 1). The centres of spheres that form these molecules are separated by a unit length (l=σ) regardless of their diameters. Consequently, spheres may either overlap or be separated by a gap depending on whether $\sigma^{\prime }$ is greater than or less than σ. This type of molecule is called a *dumbbell* (see Figure 1). The arrangement of molecules varies with the number of inclusion layers in the system. When one (001) inclusion layer is separated by matrix layers within the FCC lattice, only two orientations are possible: [110] and $\left[1\bar{1}0\right]$. However, if there are two or more adjacent inclusion layers, four more directions become available: [101], $\left[10\bar{1}\right]$, [011], and $\left[01\bar{1}\right]$.

The current simulations used representative supercells composed of $N_{x}N_{y}N_{z}$ FCC unit cells with periodic boundary conditions. Here, $N_{i}$ indicates the number of FCC unit cells along each direction. We studied a system with inclusion layers stacked periodically along the *z*-axis, extending infinitely in the OXY plane. The supercell contains $N_{inc}=2L\cdot N_{x}N_{y}$ inclusion spheres and $N_{inc}/2$ molecules, where *L* is the number of inclusion layers. The ratio $N_{inc}/N$ is called the concentration, *c*, of inclusion spheres in the system. Since $N_{inc}$ is even, the pairing of spheres into molecules is arranged so that no inclusion sphere remains unpaired. Figure 1 displays samples of all studied systems.

### 2.2. Methods

#### 2.2.1. Elastic Properties

The elastic properties of the studied systems were evaluated through Monte Carlo (MC) computer simulations. These simulations utilized the Parrinello–Rahman method [40,41,42], allowing for the calculation of the complete elastic compliance tensor within the isobaric–isothermal (NpT) ensemble. As shown in Ref. [41], the Lagrange strain tensor can be expressed in terms of matrices called the box (h) and the reference box ($h_{p}$) matrices. The strain tensor is defined in the following form:

(2) $$\varepsilon =\frac{1}{2}\left(h_{p}^{-1}.h.h.h_{p}^{-1}-I\right)\;,$$

where I is the identity matrix. The box matrix h is a symmetric matrix that represents the shape of the simulation box, composed of vectors that describe its dimensions. The reference matrix, $h_{p}$, is the average of **h** at equilibrium under the dimensionless pressure $p^{*}=p\beta \sigma^{3}$, and it is formally defined as $h_{p}\equiv \langle h\rangle$. The components of the Lagrange elastic strain tensor allow for calculation of the full elastic compliance tensor using the following formula [42]:

(3) $$S_{\alpha \beta \gamma \delta }=\beta V_{p}\langle ∆\varepsilon_{\alpha \beta }∆\varepsilon_{\gamma \delta }\rangle \;.$$

In Equation (3), $V_{p}=\left|\det\left(h_{p}\right)\right|$ is the average volume of the system at pressure $p^{*}$ and $∆\varepsilon_{\alpha \beta }=\varepsilon_{\alpha \beta }-\langle \varepsilon_{\alpha \beta }\rangle$, whereas $\langle \varepsilon_{\alpha \beta }\rangle$ is the average of the components of the strain tensor in the NpT ensemble, and α,β,γ,δ = *x*, *y*, or *z*. Using elastic constants is generally more intuitive than using elastic compliances, and elastic constants can be easily derived from the elastic compliance tensor using the following relation [43]:

(4) $$\sum_{n,m}S_{ijmn}B_{mnkl}=\frac{1}{2}\left(\delta_{ik}\delta_{jl}+\delta_{il}\delta_{jk}\right)\;,$$

where $\delta_{ij}$ is the Kronecker delta. Moreover, knowledge of the complete elastic compliance tensor enables a comprehensive description of a crystal’s elastic properties for any symmetry. In particular, Poisson’s ratio in any direction can be calculated using the following formula [44]:

(5) $$\nu_{nm}=-\frac{m_{\alpha }m_{\beta }S_{\alpha \beta \gamma \delta }n_{\gamma }n_{\delta }}{n_{\zeta }n_{\eta }S_{\zeta \eta \kappa \lambda }n_{\kappa }n_{\lambda }}\;.$$

In the equation above, PR depends on two mutually orthogonal directions, represented by unit vectors $\vec{n}$ and $\vec{m}$. The first vector points in the direction of the applied external stress, while the second ($\vec{m}$) indicates the direction in which PR is measured. Greek indices follow the Einstein summation convention. For simplicity, throughout the rest of this manuscript, tensor components such as $B_{\alpha \beta \gamma \delta }$ are expressed in terms of the elastic constants matrix $B_{ij}$ in Voigt notation [45]. Here, the Latin indices *i* and *j* range from 1 to 6, reflecting the symmetric nature of this matrix. This approach is valid for infinitesimal deformations. The dependencies of Poisson’s ratio on the elastic constants in the main and auxetic directions can be read as

(6) $$\begin{array}{ccc} \nu_{\left[100][010\right]} & = & \frac{B_{13}^{2}-B_{12}B_{33}}{B_{13}^{2}-B_{11}B_{33}}, \end{array}$$

(7) $$\begin{array}{ccc} \nu_{\left[100][001\right]} & = & \frac{B_{13}\left(B_{11}-B_{12}\right)}{-B_{13}^{2}+B_{11}B_{33}}, \end{array}$$

(8) $$\begin{array}{ccc} \nu_{\left[111\right]\left[11\bar{2}\right]} & = & -\frac{3B_{44}\left(2B_{13}^{2}-B_{33}\left(B_{11}+B_{12}-2B_{66}\right)-2B_{13}B_{66}\right)}{D_{1}}, \end{array}$$

(9) $$\begin{array}{ccc} \nu_{\left[111\right]\left[1\bar{1}0\right]} & = & \frac{N_{1}}{D_{1}}, \end{array}$$

where

$$\begin{array}{cc} N_{1}= & \left(B_{11}+B_{12}\right)B_{33}B_{44}-2B_{13}^{2}\left(B_{44}-4B_{66}\right)-10B_{13}B_{44}B_{66}\\ \; & +2B_{66}\left(B_{44}\left(2\left(B_{11}+B_{12}\right)+B_{33}\right)-2B_{33}\left(B_{11}+B_{12}\right)\right), \end{array}$$

and

$$\begin{array}{cc} D_{1}= & 2(\left(B_{11}+B_{12}\right)B_{33}B_{44}-4B_{13}B_{44}B_{66}\\ \; & +\left(2\left(B_{11}+B_{12}\right)B_{33}+\left(B_{11}+B_{12}+2B_{33}\right)B_{44}\right)B_{66}-2B_{13}^{2}\left(B_{44}+2B_{66}\right)). \end{array}$$

(10) $$\begin{array}{ccc} \nu_{\left[110][001\right]} & = & \frac{4B_{13}B_{66}}{-2B_{13}^{2}+B_{33}\left(B_{11}+B_{12}+2B_{66}\right)}, \end{array}$$

(11) $$\begin{array}{ccc} \nu_{110[1\bar{1}0]} & = & \frac{-2B_{13}^{2}+B_{33}\left(B_{11}+B_{12}-2B_{66}\right)}{-2B_{13}^{2}+B_{33}\left(B_{11}+B_{12}+2B_{66}\right)}, \end{array}$$

(12) $$\begin{array}{ccc} \nu_{\left[101\right]\left[0\bar{1}0\right]} & = & \frac{2B_{44}\left(B_{11}B_{13}-B_{13}\left(B_{12}+B_{13}\right)+B_{12}B_{33}\right)}{D_{2}}, \end{array}$$

(13) $$\begin{array}{ccc} \nu_{\left[101\right]\left[\bar{1}01\right]} & = & \frac{2\left(B_{11}-B_{12}\right)\left(B_{33}\left(B_{11}+B_{12}\right)-2B_{13}^{2}\right)}{D_{2}}-1, \end{array}$$

where

$$\begin{array}{cc} D_{2}= & \left(B_{11}-B_{12}\right)\left(B_{33}\left(B_{11}+B_{12}\right)-2B_{13}^{2}\right)\\ \; & +B_{44}\left(B_{11}^{2}-{\left(B_{12}-B_{13}\right)}^{2}+B_{11}\left(B_{33}-2B_{13}\right)\right). \end{array}$$

Using the formula (Equation (5)), one can calculate the minimum PR value for any given $\vec{n}$ direction. When sampled over a large number of different directions, one obtains a good approximation of the minimum PR surface. For partially auxetic systems, taking the negative part of this surface and plotting it in a spherical coordinate system, a certain geometrical shape is obtained. The latter shape is characteristic of the crystal’s symmetry. The volume of this shape can be used to calculate the degree of auxeticity (χ) [46]:

(14) $$\chi =\sqrt[{3}]{\frac{3A}{4\pi }}\;,$$

which is the relative measure of how strongly auxetic the system is compared to the ideal auxetic. In the above, the value *A* is defined as

(15) $$A=\int_{0}^{2\pi }d\phi \int_{0}^{\pi }\sin\theta d\theta \int_{0}^{R(\theta ,\phi )}r^{2}dr\;,$$

and $R\left(\theta ,\phi \right)={\left(2\pi \right)}^{-1}\int_{0}^{\pi }\left(\left|\nu_{n(\theta ,\phi )}\left(\alpha \right)\right|-\nu_{n(\theta ,\phi )}\left(\alpha \right)\right)d\alpha$ is the mean of negative values of Poisson’s ratio corresponding to the direction of $\vec{n}$. The angle α is the angle between $\vec{m}$ and the direction which is created by the crossing of the OXY plane and the plane orthogonal to $\vec{n}$. For more details, see Ref. [46].

#### 2.2.2. Computational Details

This study used the standard MC scheme [39], employing a Metropolis algorithm with two types of trial moves. The first adjusts particles’ positions and orientations, while the second modifies components of the symmetric box matrix (h). Both move types had about a 40% acceptance rate, but attempts to change the box matrix components were $N^{1/2}$ times less frequent than particle moves. Models with N=864 hard spheres were analyzed, representing a crystal of 6×6×6 FCC unit cells. Consequently, the inclusions contained 72, 144, and 216 spheres for systems with one, two, and three layers, respectively. The sphere inclusion concentrations were 8.33(3)%, 16.66(6)%, and 25%. All simulations were conducted at a reduced pressure of $p^{*}=50$. The effect of varying inclusion sphere diameters was studied within the $\sigma^{\prime }/\sigma$ range from 0.95σ to 1.05σ, with these values fixed during each run. Prior research [34] showed that nanolayer inclusions orthogonal to the [001]-direction in FCC crystals reduce symmetry from cubic to tetragonal. This work is focused on conditions where a stable tetragonal phase emerged at specific ($p^{*}$, $\sigma^{\prime }/\sigma$) parameters. For inclusion layers made of molecules, elastic constants were averaged over 950 simulations for each $\sigma^{\prime }/\sigma$ value. Each simulation run involved $10^{7}$ MC cycles, with the initial 10%—during which the system reached equilibrium—excluded from calculations.

## 3. Results and Discussion

In systems where particles interact via a hard potential, the elastic properties can be modified by adjusting particle sizes or shapes. Here, we further extend recent studies on model hard-sphere crystals with layered sphere inclusions [35], which also serves as a starting point for this study. In addition to altering atomic diameters, the main focus of this study is to examine how reducing the number of atomic degrees of freedom in the inclusion layers affects the system’s elastic properties. This reduction can be viewed as imposing additional constraints on the inclusion particles by bonding neighbouring hard spheres into diatomic molecules.

**Figure 2:**
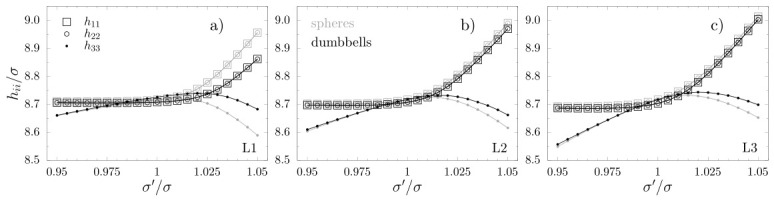
The diagonal elements of the box matrix h for systems with dumbbell (black) are compared to systems with hard-sphere (gray) inclusions as a function of $\sigma^{\prime }/\sigma$ in the case of (**a**) one, (**b**) two, and (**c**) three inclusion layers. Calculation errors do not exceed the size of the symbols.

Research shows that in crystals with inclusions orthogonal to the [001] direction, any value of $\sigma^{\prime }/\sigma \neq 1$ reduces the crystal’s symmetry from cubic to tetragonal [45]. To see if this also applies to molecular inclusions, Figure 2 compares the diagonal components of the box matrices across all studied systems, with models containing only inclusions formed by spheres. Systems with one, two, and three inclusion layers are shown in graphs (a), (b), and (c). The off-diagonal elements of h are at least four orders of magnitude smaller than the diagonal ones and are treated as zero, consistent with previous studies. It is clear that as $\sigma^{\prime }/\sigma$ increases, the shape shifts from cubic to cuboid, with in-plane directions [100] and [010] expanding, while the [001] direction contracts both for systems of spheres and dumbbells at higher $\sigma^{\prime }/\sigma$. Although this change is less pronounced for dumbbells. Contrary, when $\sigma^{\prime }/\sigma <1$, the sample compresses along the [001] direction; however, $h_{11}$ and $h_{22}$ show minimal change, as the crystal’s matrix cannot be compressed further.

**Figure 3:**
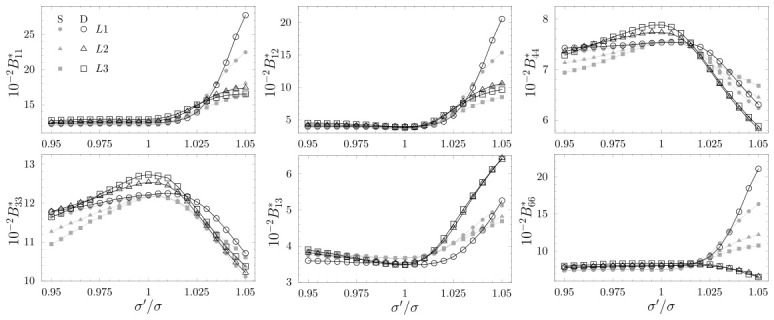
The elastic constants $B_{ij}$ are shown as a function of $\sigma^{\prime }/\sigma$ for dumbbell systems (black), which include one (L1—circles), two (L2—triangles), and three (L3—squares) inclusion layers. The results are compared to systems with inclusions made only of spheres. They are plotted with corresponding filled symbols in gray colour. The number of inclusion layers is also indicated by the size of the symbol. Calculation errors do not exceed the size of the symbols.

Figure 3 presents the elastic constants for the studied systems and compares them with systems that have only spheres as inclusions. In our case, the following equalities occur: $B_{11}=B_{22}$, $B_{13}=B_{23}$, and $B_{44}=B_{55}$. Moreover, $B_{ij}=0$ for i=1,2,3 and j=4,5,6, as well as $B_{45}=B_{46}=B_{56}=0$. This explicitly indicates the tetragonal symmetry, notably the 422 symmetry class [45]. Adding constraints, such as molecular bonds, significantly affects elastic properties. At $\sigma^{\prime }/\sigma =1$, the elastic constants for molecular systems differ from those of atomic systems, notably in $B_{33}$, $B_{13}$, and $B_{44}$. The curve arrangement in Figure 3 shows that at $\sigma^{\prime }/\sigma =1$, all atomic systems (spheres as inclusions) form a pure FCC crystal, where $B_{11}=B_{33},B_{12}=B_{13}$, and $B_{44}=B_{66}$. As the diameter of the inclusion spheres increases ($\sigma^{\prime }/\sigma >1$), the elastic constants of molecular systems tend to behave similarly to atomic systems. Specifically, $B_{11},B_{12}$, and $B_{13}$ increase with rising $\sigma^{\prime }$ and are inversely related to the number of inclusion layers. This aligns with the idea that, when inclusion particle concentration exceeds 50%, the system effectively becomes an inverted version, with matrix particles acting as smaller inclusion particles, approaching a pure FCC crystal at c=100%. A notable difference appears between systems with dumbbells and spheres in Figure 3: The $B_{66}$ constant decreases for L2 and L3 dumbbell systems as $\sigma^{\prime }/\sigma$ increases, unlike sphere-only systems and the L1 dumbbell system where $B_{66}$ increases. This can be explained by the additional constraint between neighbouring inclusion layers, since some dumbbells belong to adjacent layers. As a result, systems with two or more dumbbell-inclusion layers exhibit reduced shear resistance when stress is applied parallel to the inclusion plane. At large $\sigma^{\prime }/\sigma$, the $B_{66}$ values for L2 and L3 dumbbell systems are even lower than those of a pure FCC crystal. This is an essential qualitative difference between systems with spheres and dumbbells as inclusions, arising from the reduction in the number of degrees of freedom of the particles that compose the inclusions. It is worth emphasizing that, when $\sigma^{\prime }/\sigma =\sigma$ in dense packing (i.e., in the limit of infinite pressure), the sphere atoms form the FCC phase both in the system composed of only spheres and in the systems containing dumbbells. At lower densities (pressures), the sphere system retains the FCC structure, whereas the systems containing dumbbells deviate slightly from the FCC structure, thereby changing the values of the $B_{ij}$ moduli. The reason for these differences in the structure and properties of the system composed of only spheres and the system containing dumbbells is the limitation of the motion of the pairs of spheres forming the dumbbells, which cannot change their mutual distance. This results in a change in the effective collective interactions among the particles. In a system where the inclusions are spheres of another size, changes in elastic properties arise solely from the effective collective interactions between the inclusion and matrix spheres. When the spheres that compose the dumbbells and the matrix have the same diameter, we can see the effects of the degree-of-freedom constraint, which directly impacts the collective interactions between the dumbbells (inclusion particles) and the matrix spheres. However, when the diameter of the dumbbell spheres also varies, an additional size-related factor of dumbbells comes into play, further influencing the collective interactions between the inclusion and matrix particles.

**Figure 4:**
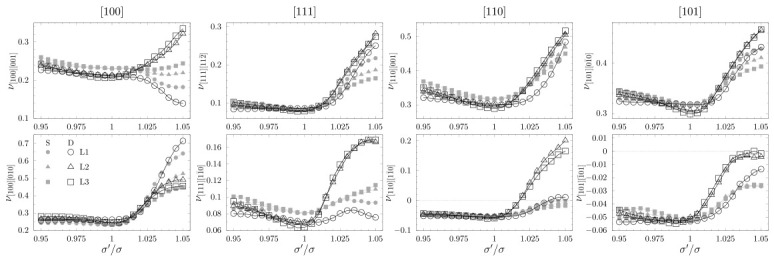
Poisson’s ratio for systems with dumbbell inclusions (open, black) compared to systems with inclusions containing only spheres (filled, gray). Systems with one (L1), two (L2), and three (L3) inclusion layers are marked with circles, triangles, and squares, respectively. Calculation errors do not exceed the size of the symbols.

In the calculations detailed in Section 2.2.1, knowing the elastic compliance matrix or the elastic constants matrix enables one to determine Poisson’s ratio in any crystallographic direction within the crystal. Figure 4 displays the PR values for four selected $\vec{n}$ directions and two $\vec{m}$ directions for each respective $\vec{n}$. As shown in Figure 4, introducing spheres as inclusions generally raises Poisson’s ratio as the sphere diameter increases. This effect is most noticeable in L1 systems and weakens with additional layers. A similar trend is observed in the dumbbell L1 system, which behaves like systems consisting only of spheres. However, in the L2 and L3 dumbbell systems, the PR increase is more pronounced in the [100][001] and $\left[111\right]\left[1\bar{1}0\right]$ directions. In the $\left[110\right]\left[1\bar{1}0\right]$ direction, the rise in $\sigma^{\prime }/\sigma$ leads to a loss of auxetic behaviour across all three dumbbell systems; additionally, the L2 and L3 systems exhibit a significant rise in Poisson’s ratio compared to L1. In the $\left[101\right]\left[\bar{1}01\right]$ direction, another auxetic direction within the FCC crystal, embedding dumbbells in the inclusion layers significantly reduces auxetic behaviour compared with the system with spheres, although the PR remains negative.

**Figure 5:**
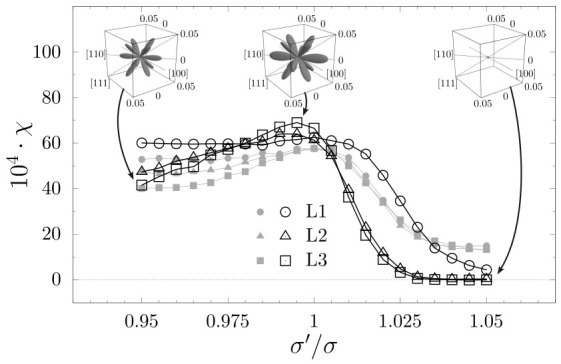
The degree of auxeticity χ for the studied systems as a function of $\sigma^{\prime }/\sigma$. Open black symbols represent results for systems with dumbbell inclusions. Results for systems with inclusions consisting solely of spheres are shown in gray filled symbols. The inserts show the absolute value of the minimum negative Poisson’s ratio across all crystallographic directions in spherical coordinates for the three-layer dumbbell inclusion system. Calculation errors do not exceed the size of the symbols.

Figure 4 presents changes only in eight crystallographic directions. To quantify the overall change in the system’s auxeticity, we have calculated the degree of auxeticity (χ) for the studied systems (Equation (14)). The results are plotted with respect to $\sigma^{\prime }/\sigma$ in Figure 5. In the figure, the maximum value of the auxeticity degree ($\chi =6.9\cdot 10^{-3}$) corresponds to the system L3 with dumbbells at $\sigma^{\prime }/\sigma$ around 0.995. This value is higher than the similar maximum for systems with hard spheres ($\chi =5.76\cdot 10^{-3}$), which appears at $\sigma^{\prime }/\sigma =1.0$. Overall, the auxetic properties of the system with dumbbell inclusions (at $\sigma^{\prime }/\sigma =0.995$) are stronger ($\chi =6.9\cdot 10^{-3}$) than those of the hard-sphere systems ($\chi =5.68\cdot 10^{-3}$). This indicates that connecting neighbouring spheres to form diatomic molecules within inclusion layers can enhance auxetic properties. This behaviour is the result of collective interactions, similarly to those considered for systems with the hard-core repulsive Yukawa potential [47], with the difference that, in this case, the auxetic enhancement is due to the ‘competition’ of particle interaction potentials resulting from the change in particle shape from spheres to dumbbells, which is associated with a reduction in the particles’ degrees of freedom. On the other hand, increasing $\sigma^{\prime }/\sigma$ strongly weakens the auxetic properties of all studied systems. Moreover, in systems with large diameters of the spheres forming dumbbells, the crystal’s auxetic properties have been almost eliminated. The three-layer dumbbell inclusion system exhibits a degree of auxeticity as low as $\chi =3.5\cdot 10^{-7}$. Within the parameter range corresponding to thermodynamically stable systems, there was no complete suppression of auxetic properties. So, we observed two opposing effects—enhancement and practical elimination of auxetic properties—that arise from ‘competition’ among intermolecular interactions mediated by particle shape and size.

In both cases, the weakening (spheres as inclusions) and near-elimination (dumbbells as inclusions) of auxetic properties result from the nonmonotonic behaviour of elastic constants $B_{33}$, $B_{44}$, and $B_{66}$ (except the $B_{66}$ for L1 dumbbell system), which reach a maximum around $\sigma^{\prime }/\sigma \approx 1$ (see Figure 3). In the case of systems with dumbbells, however, there is an additional effect from reducing the particles’ degrees of freedom, which increases the values of $B_{33}$, $B_{44}$, and $B_{66}$ near $\sigma^{\prime }/\sigma \approx 1$, making the system with dumbbells stiffer than the system with spheres in the corresponding directions. Here, the maximum values of $B_{33}$ and $B_{44}$ elastic constants for the L3 system are about 4.5% higher for the system with dumbbells compared with the system with spheres as inclusions. Later, with increasing $\sigma^{\prime }/\sigma$, the values of $B_{33}$ and $B_{44}$ for the system with dumbbells decrease faster than the system with spheres as inclusions. Finally, for $\sigma^{\prime }/\sigma =1.05$, the elastic constants $B_{33}$ and $B_{44}$ are, respectively, about 2.3% and 11.7% lower than for the system with spheres. Since, in the formula for Poisson’s ratio in auxetic directions (Equations (11) and (13)), the elastic constants $B_{33}$, $B_{44}$, and $B_{66}$ are the only nonmonotonic functions of $\sigma^{\prime }/\sigma$ and the remaining elastic constants monotonically increase with $\sigma^{\prime }/\sigma$ (see Figure 3), the auxetic properties are essentially weakened for the system with spheres and almost disappear in the case of the system with dumbbells. This follows directly from the functional relationship between the numerators and denominators of Equations (11) and (13), which arises from the nonmonotonic behaviour of $B_{33}$, $B_{44}$, and $B_{66}$ as a function of $\sigma^{\prime }/\sigma$. It is worth noting that the maximum degree of auxeticity of the system with dumbbell inclusions results from an increase in the system’s stiffness, manifested by the maximum values of $B_{33}$, $B_{44}$, and $B_{66}$ (see Figure 3), which arise from the reduction in the degrees of freedom of atoms as already mentioned above.

The inserts in Figure 5 display the absolute values of the minimum negative Poisson’s ratio in spherical coordinates for three $\sigma^{\prime }/\sigma$ values in the L3 dumbbell system. It is apparent that at high $\sigma^{\prime }/\sigma$ ratios, the auxetic behaviour diminishes across all inclusion-bearing systems, with the most notable reduction in the molecular L2 and L3 systems, where the auxetic properties are practically eliminated. This is reflected in the right insert in Figure 5. The middle insert represents the absolute value of the minimum negative Poisson’s ratio for the system with three-layer dumbbell inclusions, which corresponds to the maximum value of the degree of auxeticity and is manifested by the largest volume of the shape of the figure. The left insert represents the system with three-layer dumbbell inclusions at $\sigma^{\prime }/\sigma =0.95$, where the negative PR remains largely unchanged compared to the L3 system with sphere inclusions only.

One of the key advantages of the system with dumbbell inclusions instead of spheres is a much larger range of variation in both the total auxeticity of the system (Figure 5) and the Poisson’s ratio in the auxetic directions (Figure 4). In the case of the system containing only spheres, the degree of auxeticity varies between $1.34\cdot 10^{-3}$ and $5.76\cdot 10^{-3}$, while for the system with dumbbell inclusions, it varies between $3.52\cdot 10^{-7}$ and $6.9\cdot 10^{-3}$ (Figure 5). The range of change of Poisson’s ratio in the $\left[110\right]\left[1\bar{1}0\right]$ direction for the system of spheres is from −0.0508 to −0.017; for the L3 system with dumbbells, it is from −0.0589 to 0.165 (Figure 4); in the $\left[101\right]\left[\bar{1}01\right]$ direction for the L3 system of spheres, it is from −0.0521 to −0.0257; for the system with dumbbells, it is from −0.0548 to 0 (Figure 4).

## 4. Conclusions

The elastic properties of a hard-sphere crystal with multiple (001) nanolayer inclusions of diatomic molecules (dumbbells) were evaluated using Monte Carlo simulations in the NpT ensemble. These layer inclusions were incorporated into the FCC crystal as a periodic sequence of single or adjacent (001) crystallographic planes. The study compared the elastic behaviour of systems containing one, two, or three neighbouring dumbbell layers with that of a system containing similar nanolayer inclusions composed of spheres of a different diameter. It focused on how reducing atomic degrees of freedom within the layers (by randomly bonding neighbouring hard spheres into diatomic molecules) affects the FCC hard-sphere crystal’s auxetic properties.

Introducing nanolayers of dumbbells into a pure hard-sphere crystal significantly affects its elastic properties, especially when $\sigma^{\prime }/\sigma >1$. Studies indicate that the both types of molecules (spheres or dumbbells) within the layers, as well as the number of layers, are crucial to altering the crystal’s elasticity. The approach used to restrict atomic degrees of freedom within the layers generally increases the elastic constants compared to systems with nanolayers made of spheres of different sizes. However, for constants $B_{33}$ and $B_{44}$ in systems with dumbbell-molecule inclusions, an opposite trend is observed. The most intriguing qualitative finding is that $B_{66}$ for systems with two or three dumbbell-inclusion layers decreases with $\sigma^{\prime }/\sigma$, in contrast to the other studied systems. This difference arises from the additional constraint between neighbouring inclusion layers, since some dumbbells are oriented out-of-plane and bind spheres in different layers. Consequently, systems with two or more dumbbell-inclusion layers have lower shear resistance when stress is applied parallel to the inclusion plane. This finding provides a way to tailor elastic properties not only quantitatively but also qualitatively by changing the particle type in the includion layer, specifically by switching from spheres to dumbbells.

A notable finding concerns the auxetic properties of the studied systems. For $\sigma^{\prime }/\sigma >1$, it was observed that decreasing the atoms’ degrees of freedom by randomly linking neighbouring spheres into dumbbells nearly eliminates auxetic behaviour. This effect becomes more significant as the number of adjacent inclusion layers grows in this ratio range. The most interesting effect observed for values of $\sigma^{\prime }/\sigma$ around 1.0 is the enhancement of auxetic properties due to bonding neighbouring hard spheres into dumbbells. This enhancement arises from the ’competition’ among particle interaction potentials resulting from the change in particle shape from spheres to dumbbells, associated with the reduction in the particles’ degrees of freedom. This shows that reducing the atomic degrees of freedom within layer inclusions embedded in a hard-sphere crystal by bonding neighbouring hard spheres into dumbbells is an efficient way to alter the elastic properties of the systems, especially their auxetic behaviour.

In summary, reducing the atomic degrees of freedom in the inclusion layers can be viewed as a means of modifying the system’s auxeticity and tuning the material’s elastic properties. Notably, current nanotechnology can already produce monatomic layers [48]. Also, given the possibilities for synthesizing nanoparticles of various geometries [49,50,51], the experimental realization of systems with reduced degrees of freedom appears within reach. Metamaterials based on the presented systems can be applied directly as auxetic materials, also in acoustics, to moderate the band gap. Hence, the concepts and results from this study may inspire nanoscience researchers to develop layered structures for practical use in designing metamaterials with tailored characteristics.

It is worth noting that, although systems with a hard potential are fundamental model systems, they also have direct applications in describing colloidal systems [28]. A further extension, especially for studies of colloidal crystals, is to model systems in which particles interact via an effective screened Coulomb potential, known as the hard-core repulsive Yukawa potential, which successfully describes the purely repulsive interaction between a pair of similarly charged colloidal particles [52]. In the near future, we plan to present research results using this more realistic potential and examine its impact on elastic properties compared with the hard potential.

In closing, it is worth noting that the present study demonstrates that predicting how specific atomic-level structural modifications affect a crystal’s elastic properties is a non-trivial task. Nonetheless, computer simulations serve as a highly effective tool for this task.

## Data Availability

The original contributions presented in this study are included in the article. Further inquiries can be directed to the corresponding author.

## Abbreviations

The following abbreviations are used in this manuscript (listed in order of occurrence in the text):

PR	Poisson’s ratio
FCC	Face-centered cubic
HS	Hard sphere
MC	Monte Carlo
